# Morbidity and mortality in critically ill patients with invasive group A streptococcus infection: an observational study

**DOI:** 10.1186/s13054-020-03008-z

**Published:** 2020-06-06

**Authors:** Viveka Björck, Lisa I. Påhlman, Mikael Bodelsson, Ann-Cathrine Petersson, Thomas Kander

**Affiliations:** 1 Skåne University Hospital, Department of Clinical Sciences Lund, Anaesthesiology and Intensive Care, Lund University, SE-221 85 Lund, Sweden; 2 Skåne University Hospital, Department of Clinical Sciences Lund, Infection Medicine, Lund University, SE-221 85 Lund, Sweden; 3 Clinical Microbiology Laboratory, Region Skåne, SE-221 85 Lund, Sweden

**Keywords:** *emm*1/T1-type, Group A streptococcus, Intensive care unit, Sepsis

## Abstract

**Background:**

Group A streptococci (GAS) are known to cause serious invasive infections, but little is known about outcomes when patients with these infections are admitted to intensive care. We wanted to describe critically ill patients with severe sepsis or septic shock due to invasive GAS (iGAS) and compare them with other patients with severe sepsis or septic shock.

**Methods:**

Adult patients admitted to a general intensive care unit (ICU) in Sweden (2007–2019) were screened for severe sepsis or septic shock according to Sepsis 2 definition. Individuals with iGAS infection were identified. The outcome variables were mortality, days alive and free of vasopressors and invasive mechanical ventilation, maximum acute kidney injury score for creatinine, use of continuous renal replacement therapy and maximum Sequential Organ Failure Assessment score during the ICU stay. Age, Simplified Acute Physiology Score (SAPS 3) and iGAS were used as independent, explanatory variables in regression analysis. Cox regression was used for survival analyses.

**Results:**

iGAS was identified in 53 of 1021 (5.2%) patients. Patients with iGAS presented a lower median SAPS 3 score (62 [56–72]) vs 71 [61–81]), *p* <  0.001), had a higher frequency of cardiovascular cause of admission to the ICU (38 [72%] vs 145 [15%], *p* <  0.001) and had a higher median creatinine score (173 [100–311] vs 133 [86–208] μmol/L, *p* <  0.019). Of the GAS isolates, 50% were serotyped *emm*1/T1 and this group showed signs of more pronounced circulatory and renal failure than patients with non-*emm*1/T1 (*p* = 0.036 and *p* = 0.007, respectively). After correction for severity of illness (SAPS 3) and age, iGAS infection was associated with lower mortality risk (95% confidence interval (CI) of hazard ratio (HR) 0.204–0.746, *p* <  0.001). Morbidity analyses demonstrated that iGAS patients were more likely to develop renal failure.

**Conclusion:**

Critically ill patients with iGAS infection had a lower mortality risk but a higher degree of renal failure compared to similarly ill sepsis patients. *emm*1/T1 was found to be the most dominant serotype, and patients with *emm1*/T1 demonstrated more circulatory and renal failure than patients with other serotypes of iGAS.

## Background

Sepsis and the more severe form, septic shock, are devastating conditions with high mortality and morbidity caused by a systemic infection leading to organ dysfunction [1, 2]. A recent extensive systemic review of observational studies from North America and Europe showed that 10% of patients admitted to intensive care units (ICUs) were diagnosed with septic shock, with an ICU mortality of 38% [3]. Gram-negative bacteria are the most common group of sepsis-causing organism (62%), but the incidence of gram-positive bacteria has increased in frequency over time [4].

One important gram-positive bacterium that causes sepsis is group A streptococcus (GAS), and it is remarkable how this very common bacterium, usually causing mild diseases such as pharyngitis and impetigo, can cause invasive infections that include necrotising fasciitis and streptococcal toxic shock syndrome (STSS). From a global perspective, GAS ranks among the top 10 infectious causes of human mortality [5]. GAS strains are classified based on serological typing of the T antigen, or genetic differences in the cell surface M protein, encoded by the *emm* gene. More than 220 different *emm*-types have been described [6, 7]. M proteins are virulence factors that contribute to the massive inflammatory effect seen in sepsis via stimulation of immune cells leading to extensive cytokine release [8].

Incidences of invasive group A streptococcus (iGAS) have usually been reported to be around 6 cases per 100,000 people per year [9, 10], with a dominance of *emm*1 in around 30%. In a prospective epidemiological study of a cohort of 142 adults and children from Greece [11], it was demonstrated that *emm*1 was associated with more severe infections such as STSS and higher ICU admission rates compared to other iGAS. Another major epidemiological study from North America included 9557 cases of iGAS retrospectively (3.8 cases per 100,000 people per year), with a mortality of 11.7%, and presented the most common *emm*-type to be *emm*1 (22%) [12]. Only 13–15% of patients with iGAS have been described to develop STSS, but the mortality in this group is usually high, with a range between 23 and 44% [13].

There are many valuable studies on iGAS infections where general patients are mixed with critically ill patients [14–18]. To the best of our knowledge, there is a paucity of studies where critically ill patients with iGAS are studied as a separate cohort and compared to other critically ill patients. Therefore, we performed this observational registry study on patients with iGAS infection who had been admitted to the ICU, with the primary aim to describe these patients in detail and with the secondary aim to evaluate mortality and morbidity in this cohort as compared to other patients with severe sepsis or septic shock admitted to the ICU without iGAS infection. Our hypothesis was that patients with iGAS infection fare worse concerning both morbidity and mortality than other patients with severe sepsis or septic shock admitted to the ICU.

## Methods

### Subjects

The study was approved by the Swedish Ethical Review Authority in Lund (registration number 2014/916 and 2018/866). All participants were offered an opt-out via an advertisement in the local newspaper, and the board waived the requirement for written informed consent. The manuscript was prepared according to the STROBE guidelines for observational studies [19].

All adult sepsis patients (> 18 years old) admitted to the general tertiary, 9-bed ICU at Lund University Hospital, Sweden, between 2007 and 2019 were eligible for inclusion and were screened for severe sepsis (ICD-code R65.1) or septic shock (ICD-code R57.2) according to the Sepsis 2 definition [20]. The patients were identified using data from the Swedish Intensive Care Registry. For patients with multiple admissions with a diagnosis of severe sepsis or septic shock, only the first admission was included in the study. Baseline characteristics (such as age, gender, reason for admission, origin of admission, physiological and laboratory data), as well as outcome variables, were collected from raw data, i.e. from the electronic master chart system of the hospital (Melior, Cerner, Kansas City, MO, USA) or from the patient data-management system at the ICU (IntelliSpace Critical Care and Anaesthesia, Philips, Amsterdam, the Netherlands). Mortality data were imported from the Swedish Intensive Care Registry.

Individuals with iGAS infection were identified by cross-referencing the ICU sepsis cohort with the database for cultures at the Clinical Microbiology Laboratory, Region Skåne. IGAS infection was defined as a growth of GAS in cultures from blood or other sterile sites such as deep tissues, synovial fluids and cerebrospinal fluids. Typing of iGAS isolates was performed at the Clinical Microbiology Laboratory, Region Skåne, using T-typing (2007–2011) or *emm*-typing (2012–2019) [21–23]. The correlation between T-type and *emm*-type is complex; for example, T-type 4 correlates with *emm*-types 4, 24, 46, 60 and 63. However, T-type 1 is considered equivalent to only *emm*1 [23].

For the description of iGAS patients, medical records were manually reviewed, identifying the site of infection and other details, including the possible use of intravenous immunoglobulins (IVIG).

### Outcomes

The primary aim of the study was to describe the baseline characteristics of patients with iGAS admitted to the ICU. The secondary aim was to investigate if morbidity and mortality differed between patients with iGAS compared to patients without iGAS (controls). For these purposes, the following outcome variables were used: (1) Days alive and free (DAF) of vasopressors and invasive mechanical ventilation for the first 28 days after ICU admission. DAF has previously been extensively used to measure the degree of organ failure [24]. High numbers in DAF mean less need for organ support and lower degree of organ failure. In the present study, we used the definition of DAF without extra penalty for death. For full disclosure, the terms ventilator- and vasopressor-free days were also included. These terms include an extra penalty for death resulting in zero days alive and free if the patient dies before day 29 [24]. (2) Maximum acute kidney injury score the first 10 days after admission, according to the Acute Kidney Injury Network (AKIN) criteria (AKIN-crea). (3) Use of continuous renal replacement therapy (CRRT). (4) Maximum Sequential Organ Failure Assessment score (SOFA-max) during the ICU stay. (5) Length of ICU stay for ICU survivors. (6) Mortality (in the ICU and at 28, 90 and 180 days after admission).

### Statistical analysis

Continuous variables are presented as median (interquartile range), and all categorical variables are presented as numbers (percentage). The Mann-Whitney or Fisher’s exact test (two-tailed) was used for univariate testing of continuous and categorical variables, respectively. A two-sided *p* value of less than 0.05 was considered to indicate statistical significance.

For the secondary aim of the study, age, Simplified Acute Physiology Score (SAPS 3) [25, 26] and iGAS were used as independent, explanatory variables in all regression analysis. The survival analysis was performed using Cox regression.

The outcomes DAF ventilator, DAF vasopressor, AKIN-crea and CRRT were analysed in separate regression analysis. The distribution of DAF vasopressor and DAF ventilator was U-shaped, with patients scoring either low or high. Since this distribution pattern does not fit any commonly used regression model, we were forced to dichotomise these variables using more than 24 h of treatment as a cutoff, i.e. DAF < 27. The distribution of AKIN-crea was also U-shaped with the majority of patients with an AKIN score of 0 and was also dichotomised to no AKIN versus AKIN 1–3. Binominal variables were analysed using logistic regression. The distribution of SOFA max and length of stay did not fit any commonly used regression models and were not possible to dichotomise and were therefore not included in any regression models. The goodness of fit for all logistic regression analyses was tested using the Hosmer and Lemeshow goodness-of-fit test.

Given that only culture-positive patients were included in the iGAS group, and to investigate any interaction from the selection of control patients including also culture-negative patients, we also performed sensitivity analyses. Firstly, a comparison of the outcomes between culture-positive control patients versus other control patients was done. Secondly, new Cox regression and multivariable analyses were performed with the same variables as in the main analyses (Table 6) but only included culture-positive patients in the control group.

SPSS Statistics version 25 (SPSS Inc., Chicago, IL, USA) was used for all statistical analysis.

## Results

### Subjects

**Fig. 1:**
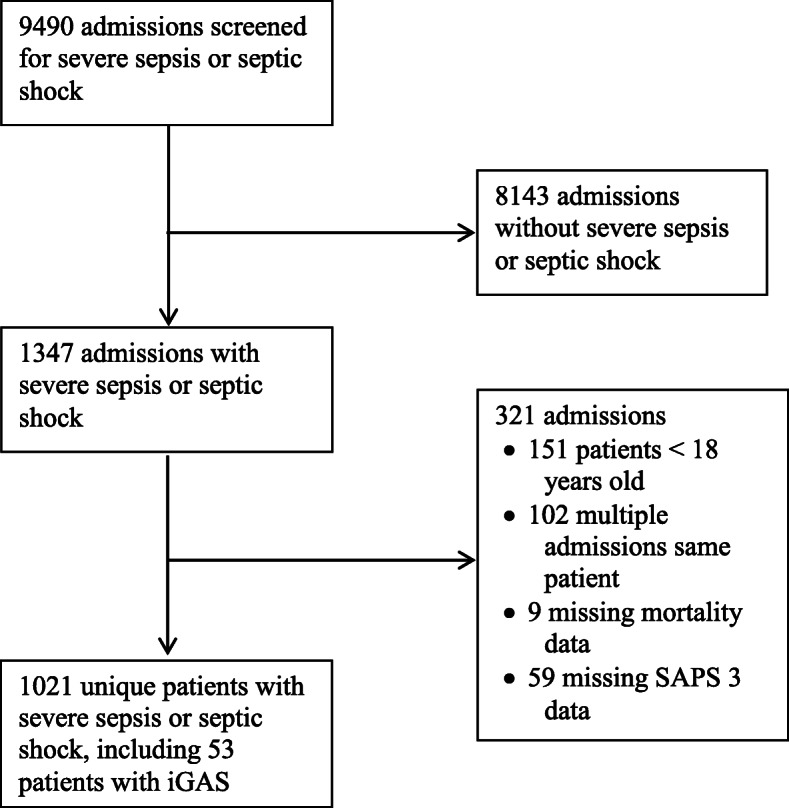
Flowchart of the patient cohort

In total, 1021 unique patients with severe sepsis or septic shock were identified out of 9490 admissions to the ICU during the study period (Fig. 1). Of these, 53 patients (5.2%) were diagnosed with iGAS infection based on growth of the bacteria in blood or from other sterile sites*.* A detailed presentation of baseline characteristics of patients with severe sepsis/septic shock, with and without iGAS, is presented in Table 1. In summary, patients with iGAS had a median age that was lower than for patients without iGAS (63 [50–70] vs 68 [59–76] years old, *p* <  0.008), presented a lower median SAPS 3 score (62 [56–72] vs 71 [61–81], *p* <  0.001) and had a higher frequency of cardiovascular cause of admission to the ICU (38 [72] vs 145 [15], *p* < 0.001), and the median creatinine score was higher (173 [100–311] vs 133 [86–208] μmol/L, *p* < 0.02). Patients with iGAS infection were less likely to be admitted from a general ward (21 [40] vs 527 [54], *p* = 0.047), and 15% arrived at the ICU from the operating room compared to 8% in the non-iGAS group (*p* = 0.074).

**Table 1:** Baseline characteristics, patients with and without invasive group A streptococcus. Values are median (Q1–Q3) or number (%)

	Non-iGAS, *n* = 968	iGAS, *n* = 53	*p* value^a^
Age (years)	68 (59–76)	63 (50–70)	0.008*
Female	421 (44)	20 (38)	0.48
SAPS 3^b^ score	71 (61–81)	62 (56–72)	< 0.001*
SAPS 3 EMR^c^ (%)	40 (21–61)	22 (14–42)	< 0.001*
Septic shock (Sepsis 3)^d^	486 (50)	32 (60)	0.16
Comorbidities			
Malignancy^e^	154 (16)	3 (5.7)	0.049*
Blood malignancy^f^	91 (9.4)	1 (1.9)	0.08
Cirrhosis^g^	30 (3.1)	3 (5.7)	0.24
Heart failure^h^	82 (8.5)	2 (3.8)	0.31
Immunosuppression^i^	105 (11)	2 (3.8)	0.11
Origin of admission			
General ward	527 (54)	21 (40)	0.047*
Emergency department	230 (24)	18 (34)	0.10
Operating room	77 (8)	8 (15)	0.074
Other ICU^j^	106 (11)	5 (9.4)	1.00
Postoperative care unit	26 (2.7)	1 (1.9)	1.00
Reason for admission^k^			
Cardiovascular^l^	145 (15)	38 (72)	< 0.001*
Hepatic	56 (5.8)	1 (1.9)	0.36
Abdominal^m^	176 (18)	9 (17)	1.00
CNS^n^	164 (17)	12 (23)	0.27
Renal	320 (33)	21 (40)	0.37
Pulmonary	206 (21)	17 (32)	0.09
Metabolic	189 (20)	9 (17)	0.72
Not coded	124 (13)	4 (7.5)	0.39
Physiological and laboratory variables at admission^o^			
Heart rate	107 (93–122)	108 (94–125)	0.85
SBP^p^ (mmHg)	103 (86–126)	104 (86–121)	0.94
Lactate (mmol/L)	2.6 (1.5–4.5)	2.5 (1.8–4.9)	0.55
Norepinephrine (μg/min/first 6 h)	2.3 (0–7.7)	2.6 (0–12)	0.10
Temperature (°C)	37.2 (36.5–38.0)	37.5 (36.9–38.0)	0.10
Leucocytes (× 10^9^/L)	11 (5.2–19)	10.5 (5.1–18)	0.78
Platelets (×10^9^/L)	160 (92–265)	163 (107–208)	0.42
pH	7.36 (7.27–7.43)	7.36 (7.29–7.42)	0.92
Bilirubin (μmol/L)	15 (9.0–26)	14 (8.0–21)	0.49
Creatinine (μmol/L)	133 (86–208)	173 (100–311)	0.02*
APTT^q^ (s)	40 (33–51)	38 (34–44)	0.19
PK-INR^r^	1.4 (1.2–1.7)	1.3 (1.2–1.4)	0.004*

^a^Fisher’s exact test or Mann-Whitney, *p ≤ 0.05

^b^Simplified Acute Physiology Score 3

^c^Estimated mortality rate

^d^All patients included in the study were diagnosed with severe sepsis or septic shock according to Sepsis 2 definition. Patients in both groups were also described as having septic shock (Sepsis 3) or not

^e^Cancer spread beyond the regional lymph nodes

^f^Lymphoma, acute leukaemia or myeloma

^g^Biopsy confirmed or clinical signs of portal hypertension

^h^NYHA class IV (fatigue, dyspnea, angina at rest)

^i^Chronic steroid treatment correlative to ≥ 0.3 mg/kg prednisolone/day, radiation or chemotherapy

^j^Intensive care unit

^k^Patients may have multiple reasons for admission

^l^Hypovolemia, cardiac shock, mixed shock, anaphylactic shock, arrhythmia or cardiac arrest

^m^Gastrointestinal bleeding, acute abdomen or pancreatitis

^n^Convulsions, decreased consciousness, coma, delirium or intracranial volume effect

^o^Blood samples taken within 90 min after admission

^p^Systolic blood pressure

^q^Activated partial thromboplastin time

^r^Prothrombin time-international normalised ratio

In the non-GAS group, culture responses from 749 patients (taken from sterile sites, including blood) were obtained. Of these, 340 (45%) were negative and 95 (12.7%) had positive cultures from more than one of the aggregated groups. For details, including bacterial species and infection sites, please see Tables 2 and 3.

**Table 2:** Culture results, in the control group

Culture result	Frequency, *n* (%)
Negative culture	340 (45)
*Escherichia coli*	98 (13)
*Staphylococcus aureus*	46 (6.1)
*Streptococcus pneumoniae*	40 (5.3)
Beta-hemolytic streptococci non-GAS	10 (1.3)
*Candida* species	20 (2.7)
*Neisseria meningitides*	2 (0.3)
*Enterococcus* species	32 (4.3)
*Pseudomonas aeruginosa*	16 (2.1)
Other gram-positive bacteria^a^	126 (17)
Other gram-negative bacteria^b^	71 (9.5)
Mixed flora^c^	3 (0.4)
*Fusarium solani*	1 (0.1)

Cultures from blood or other sterile sites from 749 of the patients in the control group. A total of 95 patients had positive cultures with microorganisms from more than one of the aggregated groups or the GAS group

^a^*Streptococcus* species (Alpha, *anginosus*, *bovis*, *intermedius*, *lutetiensis*, *mitis* and *salivarius*), Coagulase-negative *Staphylococcus (S. epidermis*, *haemolyticus*, *hominis* and *sciuri*), *Eggerthella lenta*, *Parvimonas micra*, *Bacteroides* species, *Propionibacterium*, Anaerobic gram-positive rods, *Clostridium* species *(cadaveris*, *innocuum*, *paraputrificum*, *septicum*, *ramosum*, *bifermentans* and *perfringens*), *Peptostreptococcus stomatis*, *Cutibacterium (Propionibacterium) acnes*, *Parabacteroides distasonis*, *Enterococcus gallinarum*, *Gemella* species, *Flavonifractor plautii*, *Globicatella species*, *Granulicatella* species, *Lactobacillus* species, *Anaerococcus* species, *Actinomyces odontolyticus*, *Corynebacterium* species, *Gemella morbillorum*, *Paenibacillus* species and *Peptoniphilus harei*

^b^*Proteus mirabilis*, *Klebsiella* (*aerogenes*, *oxytoca* and *pneumonia*), gram-negative rods, *Prevotella denticola*, *Serratia marcescens*, *Enterobacter cloacae*, *Dialister pneumosintes*, *Citrobacter* (*freundii* and *diversus*), *Morganella morganii*, *Salmonella enterica* serogroup *Rissen*, *Prevotella* species, *Proteus vulgaris*, *Stenotrophomonas maltophilia*, *Neisseria species*, *Haemophilus (influenza* and *parainfluenzae)*, *Sphingomonas species*, *Providencia rettgeri*, *Prevotella buccae* and *Fusobacterium necrophorum*

^c^Anaerob mixed flora, skin flora, mixed flora

**Table 3:** Infection sites, control group, *n* = 968

Pneumonia	340 (35)
Abdominal or urinary tract	259 (27)
Central nervous system	25 (2.6)
Soft tissue	19 (2.0)
Gynaecological	18 (1.9)
Other^i^	307 (32)

^i^Including but not limited to catheter-related infection, prosthesis infection and sepsis without known focus

### Results from *emm*1/T1 typing

**Fig. 2:**
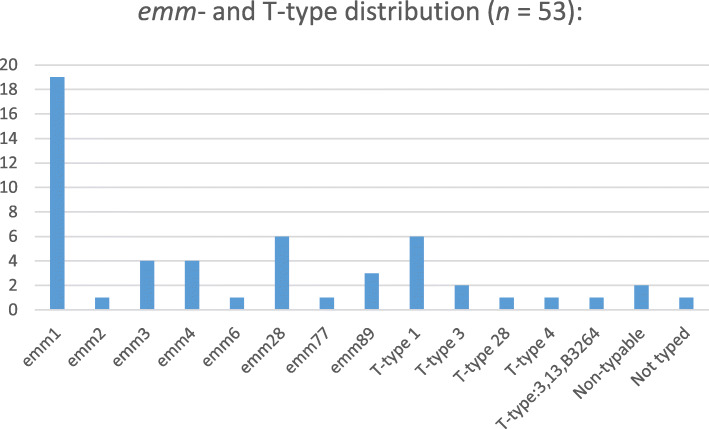
Distribution of *emm*- and T-type in iGAS isolates. Before 2012, at the Clinical Microbiology Laboratory in Lund, T-typing was performed to determine the serotype of GAS. After 2012, this was replaced by *emm*-typing. T-type 1 correlates to *emm*-type 1; other comparisons between T-type and *emm*-type are more complex

Among the 53 patients with iGAS, the isolates from one patient were not subjected to *emm*/T-typing and two isolates were non-typable. The distribution of the different *emm*-types (used after 2012) or T-types (used before 2012) is presented in Fig. 2. Of the 50 iGAS isolates with a specific *emm*/T-type, 25 isolates (50%) were classified with *emm*1/T1. Of the patients with iGAS *emm*1/T1, 72% presented with soft tissue infection compared to 44% with other *emm*/T-types (*p* = 0.08), and 48% of *emm*1/T1 had necrotising fasciitis compared to 28% in the group without *emm*1/T1 (*p* = 0.24). The incidences of septic shock (Sepsis 3 definition) and IVIG treatment were similar between *emm*1/T1 and non-*emm*1/T1 (Table 4).

**Table 4:** Patient characteristics and outcomes in iGAS patients without or with *emm*1/T1. Values are median (Q1–Q3) or number (%)

	iGAS without *emm*1/T1, *n* = 25	iGAS with *emm*1/T1, *n* = 25	*p* value^a^
Origin of infection			
Soft tissue	11 (44)	18 (72)	0.08
Necrotizing fasciitis	7 (28)	12 (48)	0.24
Pneumonia	3 (12)	3 (12)	1.00
Puerperal	2 (8.0)	0	0.49
Arthritis	4 (16)	0	0.11
Pharyngeal/parapharyngeal	1 (4.0)	2 (8.0)	1.00
Mastoiditis	1 (4.0)	0	1.00
Meningitis	0	1 (4.0)	1.00
Unknown focus	3 (12)	1 (4.0)	0.61
Septic shock (Sepsis 3)^b^	11 (44)	10 (40)	1.00
IVIG^c^	7 (28)	13 (52)	0.15
DAF^d^ vasopressor	26 (25–28)	25 (23–26)	0.036*
DAF^d^ ventilation	26 (20–28)	24 (20–26)	0.23
CRRT^e^	4 (16)	8 (32)	0.32
AKIN-crea^f^	0 (0–3)	3 (0–3)	0.007*
SOFA max^g^	9 (6–13)	12 (7–14)	0.11
Length of stay, survivors	2.7 (1.2–4.7)	5.1 (2.3–7.5)	0.08
SAPS 3^h^	65 (54–70)	61 (58–78)	0.27
EMR^i^	28 (11–39)	21 (15–54)	0.27
ICU^j^ mortality	2 (8.0)	1 (4.0)	1.00
28-day mortality	5 (20)	1 (4.0)	0.19
90-day mortality	6 (24)	2 (8.0)	0.25
180-day mortality	8 (32)	3 (12)	0.17

^a^Fisher’s exact test or Mann-Whitney, *p ≤ 0.05

^b^All patients included in the study were diagnosed with severe sepsis or septic shock according to Sepsis 2 definition. Patients in the iGAS group were also described as having septic shock (Sepsis 3) or not

^c^Intravenous immunoglobulin

^d^Days alive and free

^e^Continuous renal replacement therapy

^f^Maximum Acute Kidney Injury Network classification score the first 10 days after admission

^g^Maximum Sequential Organ Failure Assessment score during ICU admission

^h^Simplified Acute Physiology Score 3

^i^Estimated mortality rate

^j^Intensive care unit

### Outcomes

#### Non-iGAS versus iGAS patients

Outcome variables including univariate testing are presented in detail in Table 5, and results from the survival analysis and multivariable regression analysis are presented in Table 6.

**Table 5:** Outcomes with univariate testing comparing non-iGAS patients with iGAS patients. Values are median (Q1–Q3) or number (%)

	Non-iGAS, *n* = 968	iGAS, *n* = 53	*p* value^a^
DAF^b^ vasopressor	24 (25–26)	25 (22–26)	0.04*
Vasopressor free days^c^	24 (0–26)	25 (23–26)	0.027*
DAF^b^ ventilator	23 (3–28)	25 (20–28)	0.02*
Ventilator free days^c^	23 (0–28)	25 (19–28)	0.046*
CRRT^d^	185 (19)	12 (23)	0.48
AKIN-crea^e^	0 (0–3)	3 (0–3)	0.045*
SOFA max^f^	11 (8–14)	10 (6–14)	0.16
Length of stay, survivors	3.3 (1.5–6.8)	3.8 (1.8–7.0)	0.77
ICU mortality	237 (25)	3 (5.7)	< 0.001*
28-day mortality	354 (37)	7 (13)	< 0.001*
90-day mortality	429 (44)	9 (17)	< 0.001*
180-day mortality	471 (49)	12 (23)	< 0.001*

^a^Mann-Whitney or Fisher’s exact test (two-tailed)

^b^Days alive and free *without* extra penalty for death

^c^With extra penalty for death

^d^Continuous renal replacement therapy

^e^Maximal Acute Kidney Injury Network classification score the first 10 days after admission

^f^Maximal Sequential Organ Failure Assessment, score during ICU admission

**Table 6:** Associations between independent variables and outcomes. All outcomes were analysed in separate multivariable regression models as described in the “Methods” section. Morbidity outcomes were reported for the first 28 days after admission

Outcome	Age	SAPS 3^a^	iGAS
Severe sepsis or septic shock, *n* = 1021			
Mortality, CI^b^ of HR^c^	1.002–1.016*	1.032–1.044*	0.204–0.746*
DAF^d^ vasopressor, CI^b^ of OR^e^	0.994–1.015	1.044–1.07*	0.897–3.681
DAF^d^ ventilator CI^b^ of OR^e^	0.977–0.997*	1.046–1.070*	0.694–2.330
CRRT^f^, CI^b^ of OR^e^	0.979–1.002	1.031–1.054*	0.862–3.416
AKIN-crea^g^, CI^b^ of OR^e^	0.985–1.003	1.030–1.050*	1.246–4.968*

^a^Simplified Acute Physiology Score 3

^b^Confidence interval (95%)

^c^Hazard ratio

^d^Days alive and free

^e^Odds ratio

^f^Continuous renal replacement therapy

^g^Acute Kidney Injury Network (AKIN)-creatinine class 1 or worse

*p ≤ 0.05

##### Mortality

Age and high SAPS 3 correlated with higher mortality with 95% confidence interval (CI) of hazard ratio (HR 1.002–1.016, *p* < 0.05, and 1.033–1.044, *p* < 0.001, respectively). IGAS infection was associated with lower mortality risk (95% CI of HR 0.204–0.746, *p* < 0.001; Table 6). Given that *emm*1/T1 iGAS infection has been associated with more severe infections than many other iGAS serotypes [11, 12], we also performed a secondary Cox regression analysis where iGAS-serotyped *emm*1/TI was compared to the control group. The results were similar, with 95% CI of HR 0.078–0.555, *p* < 0.001, for patients with iGAS *emm*1/T1 (*n* = 25).

##### Morbidity

The goodness of fit was good with a valid chi-square value (*p* > 0.05) for all outcomes in the logistic regression analyses. As expected, an increased SAPS 3 score was associated with all measured organ failures. There was no association between any of the other independent variables included in the analysis (age and iGAS), and the development of circulatory failure measured neither with DAF vasopressors nor with CRRT (Table 6). However, higher age seemed to be associated with lower risk of respiratory failure according to DAF ventilator (95% CI of odds ratio [OR] 0.977–0.997) and there was a correlation between iGAS infection and increased risk for renal failure measured with AKIN-crea (95% CI of OR 1.266–4.034, *p* = 0.006).

#### Non-emm1/T1 versus emm1/T1

Due to the low number of patients in each group (*n* = 25 per group), it was not possible to perform multivariable regression analyses. In the uncorrected univariate analyses (Table 4), patients with *emm*1/T1 showed signs of more pronounced circulatory failure than patients with non-*emm*1/T1, measured with DAF vasopressor (*p* = 0.036). Furthermore, renal failure measured with AKIN-crea was more pronounced in the *emm*1/T1 group compared to the non-*emm*1/T1 group (*p* = 0.007). However, this was not reflected in the incidence of CRRT.

#### Sensitivity analyses

DAF ventilator was lower in the group with positive cultures compared to negative cultures (21 [2–27] vs 24 [3–28], *p* = 0.029), and the length of stay for survivors was longer in the group without positive cultures (3.3 [1.2–7.1] vs 2.6 [1.1–6.2], *p* = 0.046). All other outcomes were without differences between the groups in the univariable analysis. In the Cox regression and multivariable, only including the group with positive cultures, the results were essentially the same as in the main analysis (Additional file 1).

## Discussion

In this single-centre retrospective registry study on critically ill patients with severe sepsis or septic shock, we identified 53 unique patients with iGAS over a 12-year period. Patients with iGAS had a lower median age than the non-iGAS patients, presented a lower median SAPS 3 score at admission and had a higher incidence of cardiovascular cause for admission. After correction for severity of illness and age, iGAS infection was associated with lower mortality risk. Morbidity analyses, also corrected for severity of illness and age, demonstrated that patients with iGAS infection were more likely to develop renal failure measured with AKIN-crea.

Our hypothesis that patients with iGAS infection would fare worse concerning both morbidity and mortality compared to controls was proven wrong with regard to mortality and proven right in one aspect with regard to morbidity, i.e. renal failure. These are unexpected findings because patients with iGAS infection in general, and those presenting the *emm*1/T1 antigen in particular, have previously been described as having worse survival rates [18, 27, 28]. However, it should be noted that these studies were performed in cohorts of general patients and not only in critically ill patients, as in the present study. Furthermore, the control group in the present study included only patients with severe sepsis and septic shock, i.e. a control group with severely ill patients. Beyond that, we suggest at least two explanations for our findings. Firstly, iGAS infections are widely recognised as aggressive acute conditions where surgical treatment must be initiated without delay. This surgical treatment is normally very effective as source control and is also complemented with necessary pharmacological treatment with antibiotics and sometimes IVIG. In contrast, patients in the control group were very heterogeneous and source control is rarely as straightforward and effective as with iGAS. Regression analyses were not corrected for the fast and effective treatment in the iGAS group, which may represent a bias in the analyses. Secondly, SAPS 3 may not be sensitive enough to describe the true difference of severity of illness between the groups. As an example, it can be mentioned that comorbidity must be very severe to affect the SAPS 3 score. Considering the higher median age of patients in the control group, it is possible that patients in the control group were more severely ill than SAPS 3 will reflect. In summary, the fast and effective source control in the iGAS group, together with possible underestimated severity of illness in the control group, may contribute to the unexpected results in the corrected regression analyses.

It can be argued that the comparison between only culture-positive patients in the iGAS group with a mixture of culture-positive and culture-negative patients in the control group is unfair. The sensitivity analyses that were performed to test if this imbalance affected the main results demonstrated that it did not which indicates that this imbalance between groups did not explain the results (Additional file 1).

Although studies on critically ill patients with iGAS in the ICU are scarce, studies on all patients admitted to a hospital with iGAS are more common. Mortality in all patients with GAS infection has previously been reported to be 8–23% in the first 7 days [7, 29]. Two studies have reported mortality rates of 38–40% in patients with iGAS admitted to the ICU [30, 31]. However, in Stockmann and colleagues’ large epidemiological study on ICU patients with iGAS infection in Utah, including an impressive 1514 patients over 8 years (2002–2010), they found a mortality rate of 6% in iGAS patients > 18 years old admitted to the ICU [9]. This is in agreement with the present study where ICU mortality was 5.7% for iGAS patients (Table 5). Based on aggregated reports from the Public Health Agency in the region in which we performed our study, and given the catchment of 335,000 inhabitants for the University Hospital in Lund, the incidence of iGAS in our material was estimated at 6.0 per 100,000 inhabitants, which is in agreement with the study from Utah where the incidence was 6.3 per 100,000 inhabitants. Furthermore, in the study from Utah, the proportion of patients with iGAS infection admitted to ICU was 19%, compared to an estimated 18% (53 per 295) in the present study.

In the present study, the incidence of renal failure during the ICU stay was high in the iGAS group. The reasons for acute kidney injury (AKI) in septic patients are multifactorial. Disturbed microcirculation is considered to play an important role, since AKI in sepsis can develop in the presence of normal renal blood flow [32]. Overproduction of reactive oxygen, nitrogen species and cytokines that lead to downregulation of cell function to minimise energy demand, and thereby improving cell survival of tubular cells, are other mechanisms [32, 33]. M1 protein, situated on the surface of GAS, is a known virulence factor that leads to extensive cytokine release from monocytes and endothelial cells [8]. A rare form of acute interstitial nephritis (AIN) has also been described, where the virulence factor streptococcal pyrogenic exotoxin B (SPE B) seems to induce tubule-interstitial damage via T cell proliferation and cytokine production [34]. All this indicates that the renal failure in iGAS infection may be due to the bacteria and the immunological response induced, rather than diminished blood flow as a consequence of the hypotension in sepsis/septic shock. This may, at least in part, explain why patients in the iGAS group developed a higher degree of renal failure measured with AKIN-crea and were still more likely to survive.

A notable finding in our study is that only 50% of patients in the non-iGAS group, and 60% in the iGAS group, were diagnosed with septic shock according to the Sepsis 3 definition. In a study from 2017, Sterling and colleagues reported that in a cohort of 470 patients diagnosed with septic shock using older definitions, only 43% had septic shock according to Sepsis 3. As expected, the mortality in the two different groups differed (29% in the group meeting Sepsis 3 criteria compared to 14% using the older definition) [35]. In a large review and meta-analysis performed by Vincent and colleagues, the overall pooled frequency of septic shock diagnosed at ICU admission was 10% according to Sepsis 2 but decreased to 6.5% using Sepsis 3 criteria [3]. Taken together, this points out that Sepsis 2 overestimates the incidence of septic shock compared to Sepsis 3, which is also confirmed in our data.

Fifty patients with iGAS were typed regarding *emm*/T-type. Of these, 50% were typed as *emm*1 or T1. This is in agreement with the distribution of *emm*1 during the years with peak incidences reported from the Public Health Agency of Sweden. In 2017–2018, the incidence of iGAS in Sweden was 7.9 per 100,000 people, with a 30-day mortality of 12%. The most frequent types were *emm*1 (48%), 3, 4, 12, 28 and 89 [10]. In 2012–2013, there was also a peak in the incidence of iGAS (7.8 per 100,000) with *emm*1 (42%) dominating. The years between 2013 and 2017 reported an incidence of 5.8–6.6 per 100,000 and an *emm*1 frequency between 20 and 32% [10]. This indicates that there is a variation over time of the *emm-*types and that *emm*1 is responsible for the peak in incidences.

In our material, the majority of patients with necrotising fasciitis were found in the *emm*1/T1 group (72% vs 44% in the non-*emm*1/T1, *p* = 0.08). The severity of the infections in the *emm*1/T1 group was also underlined by a lower DAF vasopressor and higher AKIN-crea in relation to non-*emm*1/T1. There was, however, no difference in mortality regarding *emm*/T-type. This might be explained by the possibility of achieving easier source control by interventions in the operating room regarding the soft tissue infections more common in the *emm*1/T1 group, in addition to correct antibiotics and in some cases IVIG.

We recognise the limitations of the present study due to its retrospective nature. It should be noted that as in every study based on results from cultures from sterile sites, there is a risk of false-negative cultures, for example, due to cultures taken after the first dose of antibiotics. Another aspect that should be taken into consideration is that in the multivariable logistic regression analysis, higher age seemed to be associated with lower risk of respiratory failure. This result is not in agreement with the other findings in this study and the reason remains unexplained but may represent a statistical type I error. Furthermore, the number of iGAS patients is rather limited and collected from a single centre, which may not give the study sufficient power for risk prediction of all outcomes and may also question the external validity of the results.

## Conclusions

We identified 53 unique patients with iGAS during the study period of 12 years, in a large cohort of 1021 critically ill patients with severe sepsis or septic shock. *emm*1/T1 was found to be the most dominant serotype, and patients with iGAS *emm*1/T1 demonstrated more renal and circulatory failure compared to patients with iGAS infection caused by other serotypes. When comparing to a control group with substantial severity of illness, patients with iGAS infection demonstrated lower mortality risk.

## Supplementary information

**Additional file 1.** Sensitivity analyses of the control group.

## Data Availability

The datasets used and/or analysed during the current study are available from the corresponding author on reasonable request.

## Abbreviations

AIN: Acute interstitial nephritis
AKI: Acute kidney injury
AKIN: Acute Kidney Injury Network
CI: Confidence interval
CRRT: Continuous renal replacement therapy
DAF: Days alive and free
GAS: Group A streptococcus
HR: Hazard ratio
ICU: Intensive care unit
iGAS: Invasive group A streptococcus
IVIG: Intravenous immunoglobulins
SAPS 3: Simplified Acute Physiology Score 3
SOFA: Sequential Organ Failure Assessment
STSS: Streptococcal toxic shock syndrome
