# Supplementary material for: Morbidity and mortality in critically ill patients with invasive group A streptococcus infection: an observational study
Source: Crit Care. 2020 Jun 6;24:302. doi: 10.1186/s13054-020-03008-z (PMC7275847; doi:10.1186/s13054-020-03008-z)
Supplement: Supplementary file 1 — Additional file 1. Sensitivity analyses of the control group. [file 13054_2020_3008_MOESM1_ESM.docx]

**Additional file: Sensitivity analyses of the control group**

**Outcome with univariate testing comparing positive culture vs negative culture in the control group.**

Values are median (Q1-Q3) or number (%)

|  | Negative culture  (*n* = 600) | Positive culture  (*n* = 368) | *p*-value^a^ |
| --- | --- | --- | --- |
| DAF^b^ vasopressor | 24 (5-26) | 24 (4-27) | 0.63 |
| Vasopressor free days | 24 (0-26) | 24 (1-27) | 0.50 |
| DAF^b^ ventilator | 21 (2-27) | 24 (3-28) | 0.029 |
| Ventilator free days | 21 (0-27) | 24 (1-28) | 0.032 |
| CRRT^c^ | 115 (19) | 70 (19) | 1.0 |
| AKIN-crea^d^ | 0 (0-3) | 0 (0-3) | 0.82 |
| SOFA max^e^ | 10 (8-14) | 11 (8-14) | 0.24 |
| Length of stay: survivors | 3.3 (1.2-7.1) | 2.6 (1.1-6.2) | 0.046 |
| ICU^f^ mortality | 148 (25) | 89 (24) | 0.88 |
| 28-day mortality | 220 (37) | 134 (36) | 0.95 |
| 90-day mortality | 260 (43) | 169 (46) | 0.46 |
| 180-day mortality | 286 (48) | 185 (50) | 0.47 |

^a^ = Mann-Whitney or Fisher’s exact test (two-tailed)

^b^ = Days alive and free

^c^ = Continuous renal replacement therapy

^d^ = Maximum acute kidney injury network classification score the first 10 days after admission

^e^ = Maximum sequential organ failure assessment score during ICU admission

^f^ = Intensive care unit

**Associations between independent variables and outcomes using only culture positive in the control group.**

All outcomes were analysed in separate multivariable regression models as described in the Methods section. Morbidity outcomes were reported for the first 28 days after admission. * = *p* ≤ 0.05.

| **Outcome** | Age | SAPS 3 | iGAS |
| --- | --- | --- | --- |
| **Severe sepsis or septic shock, *n* = 421** |  |  |  |
| Mortality, CI of HR | 1.005 – 1.025^a^ | 1.028 - 1.047 ^a^ | 0.198 - 0.634^a^ |
| DAF vasopressor, CI of OR | 0.999– 1.031 | 1.031-1.072 ^a^ | 0.909 - 4.057 |
| DAF ventilator CI of OR | 0.970 – 0.999^a^ | 1.037-1.073 ^a^ | 0.848 – 3.010 |
| CRRT, CI of OR | 0.979 – 1.002 | 1.031-1.054^a^ | 0.862 - 3.416 |
| AKIN-crea^b^, CI of OR | 0.982 – 1.010 | 1.024-1.055 ^a^ | 1.260 – 4.289^a^ |
| ^a^ = Simplified acute physiology score 3  ^b^ = Confidence interval (95%)  ^c^ = Hazard ratio  ^d^ = Days alive and free  ^e^ = Odds ratio  ^f^ = Continuous renal replacement therapy  ^g^ = Acute kidney injury network (AKIN)-creatinine class 1 or worse |  |  |  |
